# Analysis of Cerebrospinal Fluid Extracellular Vesicles by Proximity Extension Assay: A Comparative Study of Four Isolation Kits

**DOI:** 10.3390/ijms21249425

**Published:** 2020-12-10

**Authors:** Sebastian Sjoqvist, Kentaro Otake, Yoshihiko Hirozane

**Affiliations:** Neuroscience Drug Discovery Unit, Takeda Pharmaceutical Company Limited, 26-1, Muraoka-Higashi 2-chome, Fujisawa, Kanagawa 251-8555, Japan; kentarou.ootake@takeda.com (K.O.); yoshihiko.hirozane@takeda.com (Y.H.)

**Keywords:** extracellular vesicles, cerebrospinal fluid, proximity extension assay

## Abstract

There is a lack of reliable biomarkers for disorders of the central nervous system (CNS), and diagnostics still heavily rely on symptoms that are both subjective and difficult to quantify. The cerebrospinal fluid (CSF) is a promising source of biomarkers due to its close connection to the CNS. Extracellular vesicles are actively secreted by cells, and proteomic analysis of CSF extracellular vesicles (EVs) and their molecular composition likely reflects changes in the CNS to a higher extent compared with total CSF, especially in the case of neuroinflammation, which could increase blood–brain barrier permeability and cause an influx of plasma proteins into the CSF. We used proximity extension assay for proteomic analysis due to its high sensitivity. We believe that this methodology could be useful for de novo biomarker discovery for several CNS diseases. We compared four commercially available kits for EV isolation: MagCapture and ExoIntact (based on magnetic beads), EVSecond L70 (size-exclusion chromatography), and exoEasy (membrane affinity). The isolated EVs were characterized by nanoparticle tracking analysis, ELISA (CD63, CD81 and albumin), and proximity extension assay (PEA) using two different panels, each consisting of 92 markers. The exoEasy samples did not pass the built-in quality controls and were excluded from downstream analysis. The number of detectable proteins in the ExoIntact samples was considerably higher (~150% for the cardiovascular III panel and ~320% for the cell regulation panel) compared with other groups. ExoIntact also showed the highest intersample correlation with an average Pearson’s correlation coefficient of 0.991 compared with 0.985 and 0.927 for MagCapture and EVSecond, respectively. The median coefficient of variation was 5%, 8%, and 22% for ExoIntact, MagCapture, and EVSecond, respectively. Comparing total CSF and ExoIntact samples revealed 70 differentially expressed proteins in the cardiovascular III panel and 17 in the cell regulation panel. To our knowledge, this is the first time that CSF EVs were analyzed by PEA. In conclusion, analysis of CSF EVs by PEA is feasible, and different isolation kits give distinct results, with ExoIntact showing the highest number of identified proteins with the lowest variability.

## 1. Introduction

There is a great need for CNS biomarkers for diagnostic, monitoring, and patient stratification applications. Current clinical practice largely relies on assessment of patient-reported symptoms, which is highly subjective and difficult to quantify. Novel biomarkers that could identify CNS disorders before symptom onset have the potential to greatly improve treatment of many diseases [1].

Cerebrospinal fluid (CSF) holds great promise as a source for CNS biomarkers, and its analysis is already used widely in clinical decision-making [2]. For example, measurements of AMYLOID BETA (Aβ)1–42, total TAU (T-TAU), phosphorylated TAU, and NfL have considerable clinical significance for the diagnosis of Alzheimer’s disease [3]. The concentration of GLIAL FIBRILLARY ACIDIC PROTEIN (GFAP) in CSF has been proposed as a biomarker for sports-related traumatic brain injury [4]. The ratio of CSF-to-plasma concentration of ALBUMIN can be utilized as a marker for blood–brain barrier disruption, which is of relevance to mild traumatic brain injuries [5]. CSF ALPHA-SYNUCLEIN and phosphorylated TAU are both decreased in Parkinson’s patients compared with controls [6]. The CSF levels of acute phase factors such as INTERLEUKIN-6 correlate to lesion size after ischemic stroke [7]. Without a doubt, CSF is an important source for biomarkers for a large number of CNS disorders.

One challenge when searching for biomarkers in CSF (as with most body fluids) is that highly abundant proteins such as ALBUMIN can hamper the detection and quantification of less abundant markers [1]. Another concern is that neuro-inflammation, which occurs to varying degrees in several CNS disorders, can affect blood–brain barrier (BBB) integrity and cause increased leakage of plasma proteins into the CSF [8].

Extracellular vesicles (EVs) are small, membrane-enclosed particles that likely are released by all cells [9,10]. EVs have great potential for biomarker discovery, given that their composition reflects that of their parent cells [11,12]. Using CSF-derived EVs (CSF EVs) instead of total CSF could have benefits, such as the reduction of both abundant proteins (e.g., ALBUMIN) and leaked proteins due to a loss of integrity of the BBB. CSF EVs have shown promise for biomarker discovery for diseases such as multiple sclerosis [13], CNS injury related to HIV [11], and Alzheimer’s disease (AD) [14]. One concern regarding CSF EVs is that both the amount of EVs that can be isolated per milliliter of CSF and the volume of CSF that can be acquired from one patient are limited. Although comprehensive protein analysis based on mass spectrometry has been successfully applied to CSF EV samples, a volume of eight milliliters was needed and the authors used pooled samples [15]. Acquiring such a volume from one patient is possible, although it might be challenging and analysis of smaller volumes is preferred, especially when large patient cohorts are needed. For this reason, we decided to investigate CSF EVs by the highly sensitive proximity extension assay (PEA).

PEA is not a comprehensive proteomic analysis but relies on matched antibodies labelled with partially complementary DNA oligonucleotides. Once two matching oligonucleotides are in proximity, they hybridize and are extended by DNA polymerase. These assays are performed on the microfluidic nanoliter-sized chambers. This methodology enables detection of 92 markers from one microliter of plasma. PEA has been applied to EVs derived from various cell cultures, breast milk, and seminal plasma [16]. Further, PEA has been used for biomarker discovery related to sports-related brain trauma [17], chronic mild traumatic brain injury [18], and Duchenne muscular dystrophy [19], and could also distinguish patients with Parkinson’s disease from patients with atypical parkinsonisms [20].

In this study, we included two PEA panels: the “cardiovascular III” panel, which contained several markers that potentially are important for amyotrophic lateral sclerosis (ALS), for example, YKL40/CHI3L1 [21], MCP-1 [22], and CHIT1 [23]; and the “cell regulation” panel, which included C-JUN (relevant for motor neuron degeneration [24]), ARYLSULFATASE B [25], and STX6 and STX16 (related to neuronal differentiation [26]).

It has been demonstrated that different isolation methods yield considerably different EV isolates in terms of total protein, purity, and results in downstream applications [27]. The choice of method is application-dependent. Since there were no previous reports on CSF EVs and PEA, it was unclear which EV isolation method was most suitable, and we decided to evaluate four commercially available kits. The MagCapture Exosome Isolation kit and ExoIntact both use magnetic-bead-based EV isolation, but with different EV-binding molecules. EVSecond L70 uses gravity-driven size-exclusion chromatography, while exoEasy uses membrane affinity columns. We decided to analyze EVs from one mL of CSF using PEA (cell regulation and cardiovascular III panels), as we considered one mL a feasible volume to obtain from patients.

**Figure 1:**
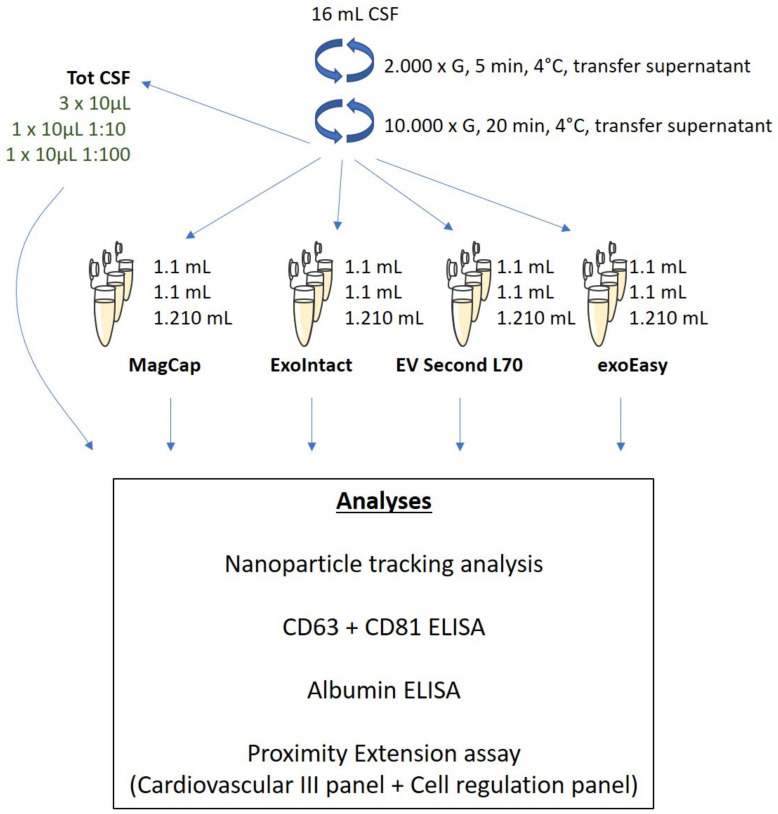
Study overview. Pooled cerebrospinal fluid (CSF) from healthy volunteers was cleared using sequential centrifugation. Extracellular vesicles (EVs) were isolated using four different commercially available kits. The isolated EVs were characterized by nanoparticle tracking analysis, ELISA, and proximity extension assay. Tot CSF: total CSF, MagCap: MagCapture.

The study overview is depicted in Figure 1. Pooled CSF was cleared using differential centrifugation, and aliquots were saved as “total CSF” samples. Three aliquots were prepared for each kit (one with a larger volume to enable 1:10 and 1:100 dilutions), and EVs were isolated as technical triplicates (three isolations per kit). The samples were analyzed by nanoparticle tracking analysis and ELISA for CD63, CD81, and ALBUMIN. Finally, the samples were analyzed using PEA by Olink Inc using two different panels (cardiovascular III and cell regulation).

## 2. Results

**Figure 2:**
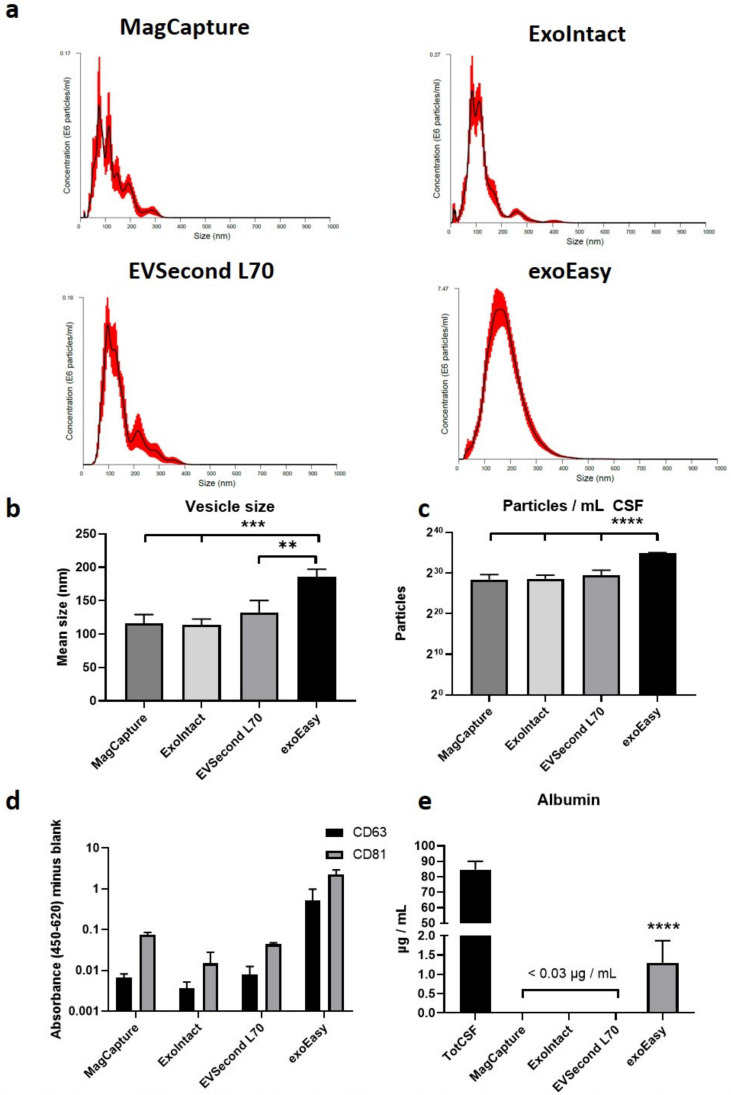
Characterization of extracellular vesicles. Nanoparticle tracking analysis was performed on samples isolated from each kit. (**a**) Representative histograms from each group. (**b**) The vesicle size was significantly larger for exoEasy samples than the other groups. (**c**) The number of isolated particles was considerably higher for exoEasy compared with the other groups. (**d**) EV markers CD63 and CD81 were detected in all groups (not measured quantitively). (**e**) Albumin, a common contaminant in EV preparations, was not detectable for MagCapture, ExoIntact, and EVSecond L70, and was heavily reduced in the exoEasy group. Significance was determined by one-way analysis of variance with Tukey’s multiple comparison tests, and labelled as ** *p* ≤ 0.01, *** *p* ≤ 0.001 and **** *p* ≤ 0.0001. Tot CSF: total CSF.

The vesicle size distribution was determined by nanoparticle tracking analysis (Figure 2a). The mean vesicle size was significantly larger for exoEasy (~185 nm), compared with the other groups (~114–133 nm) (Figure 2b). Similarly, the number of particles isolated per milliliter of CSF was significantly higher for exoEasy than for the other kits (~3 × 10^10^ vs. 3–8 × 10^8^) (Figure 2c). The EV markers CD63 and CD81 were detected in all samples (Figure 2d). Albumin, a common contaminant in EV preparations, was not detectable (less than 30 ng/mL) in the MagCapture, ExoIntact, and EVSecond L70 samples (Figure 2e). The exoEasy samples contained about 1.3 µg/mL, a reduction of over 98% compared with total CSF (~85 µg/mL).

**Figure 3:**
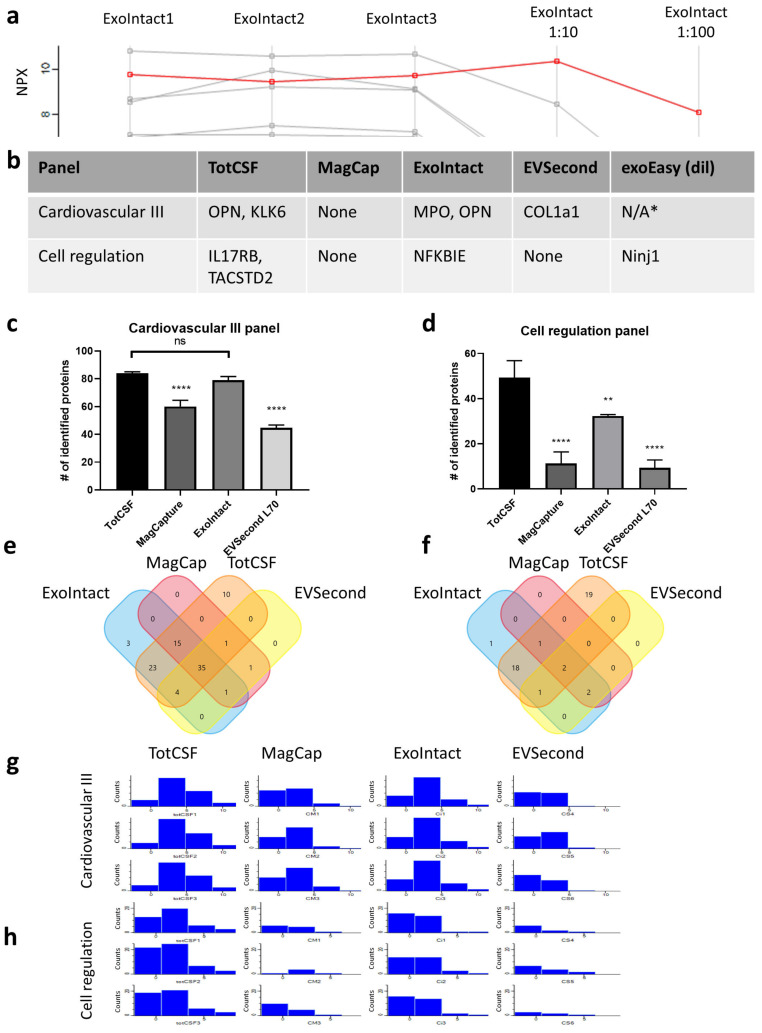
Hook analysis and protein abundance distribution. (**a**) Profile plot indicating one protein that was excluded due to a hook effect (OPN marked in red). (**b**) List of analytes that showed a hook effect from each panel and group; * indicates samples that could not be analyzed due to not meeting quality control criteria. (**c**,**d**) Number of proteins identified in each group in the cardiovascular III panel (**c**) and in the cell regulation panel (**d**). (**e**,**f**) Venn diagram of proteins detected in all three replicates of each group for cardiovascular III (**e**) and cell regulation panels (**f**). (**g**,**h**) Distribution histograms for each sample, showing the signal strength on the horizontal axis and the number of detected proteins on the vertical axis for the cardiovascular III (**g**) and cell regulation panels (**h**). The median coefficient of variation was calculated using markers with 100% detection rate in all groups. Bar charts represent *n* = 3; significance determined by one-way analysis of variance with Tukey’s multiple comparison, compared to TotCSF samples. Significance was labelled as ns for not significant, * *p* ≤ 0.05, ** *p* ≤ 0.01 and **** *p* ≤ 0.0001. Tot CSF: total CSF, MagCap: MagCapture, dil: diluted.

PEA, like other assays based on sandwich immunoassays, can be subjected to a so-called “high-dose hook effect” [28] if the concentration of antigen is in excess of the concentration of antibodies. To avoid misinterpretation of data due to a hook effect, we removed analytes that showed a signal strength of 80% or higher, compared with non-diluted samples, in either 10- or 100-fold diluted samples. Figure 3a shows two examples of a hook effect in the total CSF samples in the cardiovascular III panel (OPN and KLK6). In total, eight analytes showed a hook effect and were removed from downstream analysis (Figure 3b).

The mean number of detected proteins in the cardiovascular III panel was 84, 60, 79, and 44 for total CSF, MagCapture, ExoIntact, and EVSecond L70, respectively (Figure 3c). For the cell regulation panel, the corresponding numbers were 49, 11, 32, and 9 (Figure 3d). Thirty-five and two proteins were detected in all groups using the cardiovascular III and cell regulation panels, respectively (Figure 3e,f). The distribution histograms show the signal strength (log_2_ values) and the number of identified proteins in each bin; both panels indicate similar results—more identified proteins and a higher signal were obtained in the ExoIntact group compared with the other EV groups (Figure 3g,h).

**Figure 4:**
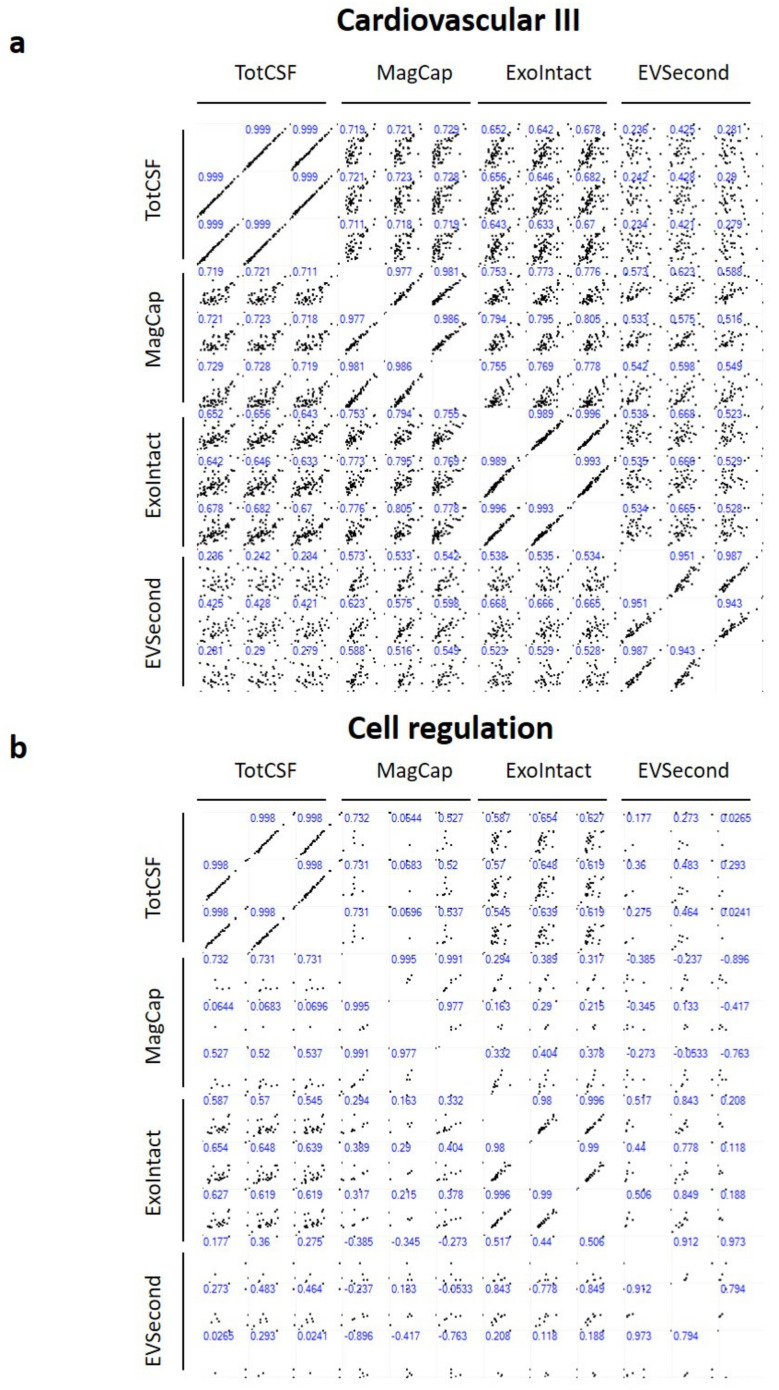
Multiscatter plot of total CSF and EV samples. Values of individual proteins in two samples are plotted for pairwise comparison (the vertical axis is labeled on the left and the horizontal axis is labeled on top). The values in blue represent Pearson’s correlation coefficients for each comparison. Data from the cardiovascular III (**a**) and cell regulation (**b**) panels are shown. Tot CSF: total CSF, MagCap: MagCapture.

The multi scatter plot for the cardiovascular III panel shows a strong correlation within the total CSF group, with a Pearson’s correlation coefficient of ~0.99 (Figure 4a). Comparing total CSF to the EV groups, the correlation was highest for MagCap and lowest for EVSecond. The correlation coefficient within each group was, on average, 0.98, 0.99, and 0.96 for MagCap, ExoIntact, and EVSecond, respectively. The correlations between either MagCap or ExoIntact and EVSecond were lower. The same trend was observed for the cell regulation panel, with ExoIntact showing the highest intragroup correlation (Figure 4b).

**Figure 5:**
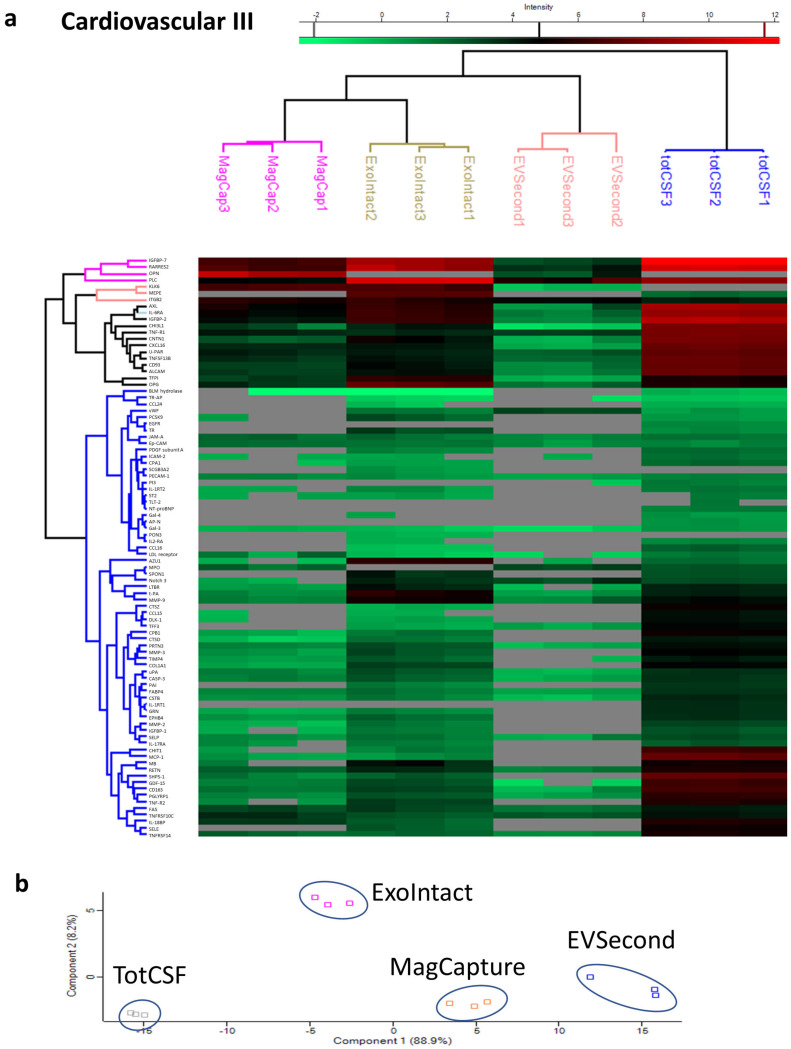
Hierarchical clustering and principal component analysis of data from the cardiovascular III panel. (**a**) Hierarchical clustering where each column represents a sample, and each row represents an analyte. The color of each cell represents intensity, as indicated by the color gradient on the top of the image; gray color indicates a missing value. (**b**) The principal component analysis (PCA) plot represents 31 proteins that were detected in all samples, showing clear clustering of samples from the different groups. Tot CSF: total CSF, MagCap: MagCapture.

**Figure 6:**
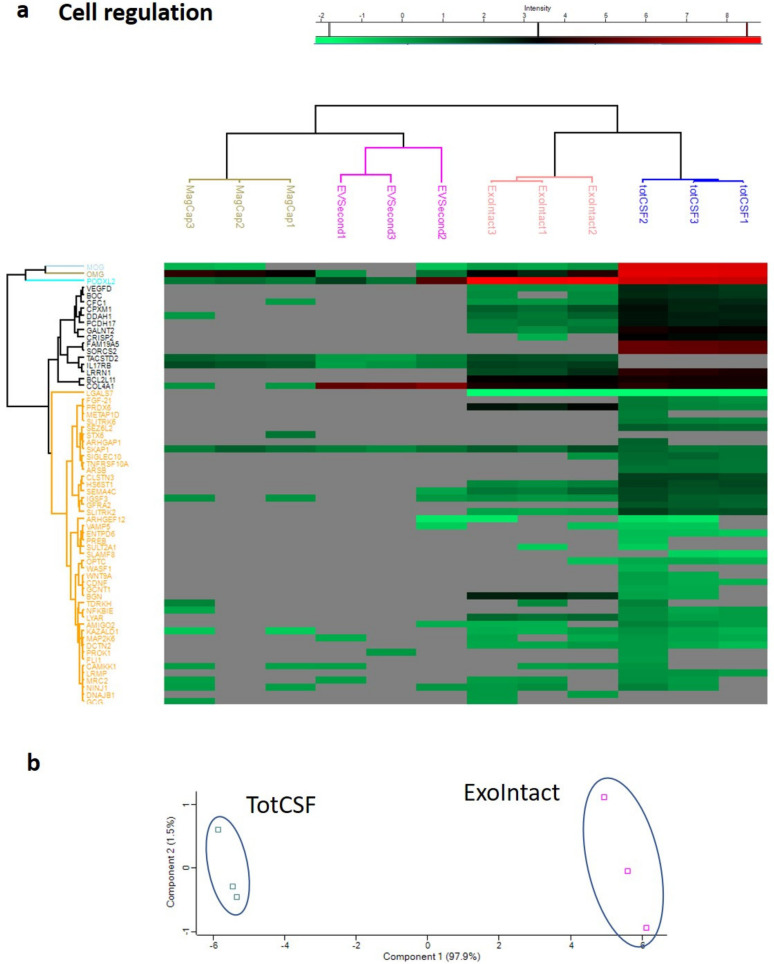
Hierarchical clustering and principal component analysis of data from the cell regulation panel. (**a**) Hierarchical clustering, where each column represents a sample, and each row represents an analyte. The color of each cell represents intensity, as indicated by the color gradient on the top of the image; gray color indicates a missing value. (**b**) The PCA plot represents 22 proteins that were detected in all samples, showing clear clustering of samples from the different groups. Tot CSF: total CSF.

We next performed hierarchical clustering with Euclidean distances with five clusters produced for rows and columns. The unsupervised clustering using the whole dataset from the cardiovascular III panel successfully clustered the samples depending on isolation kit (Figure 5a). Considering that the clustering could be a result of differences in the number of detectable proteins rather than the difference in signal strength, we performed a second clustering analysis using only proteins detected in all samples. In addition, in this analysis, the different isolation kits could successfully be clustered together (Supplementary Figure S1). We next performed principal component analysis (PCA) based on analytes that were detected in all samples (*n* = 31). PCA could clearly separate the different groups, indicating differences in the EV isolation methods (Figure 5b). The same analysis was performed for the cell regulation panel data. There were considerably fewer identified proteins. The unsupervised hierarchical clustering separated the samples into each respective group (Figure 6a). Hierarchical clustering using only proteins detected in all groups is not reported since only two markers were detected in all groups (PODXL2 and SKAP1). Consequently, PCA was only performed for ExoIntact and total CSF samples. The analysis was performed with the 22 proteins identified in both groups, and there was a clear separation of the samples (Figure 6b).

**Figure 7:**
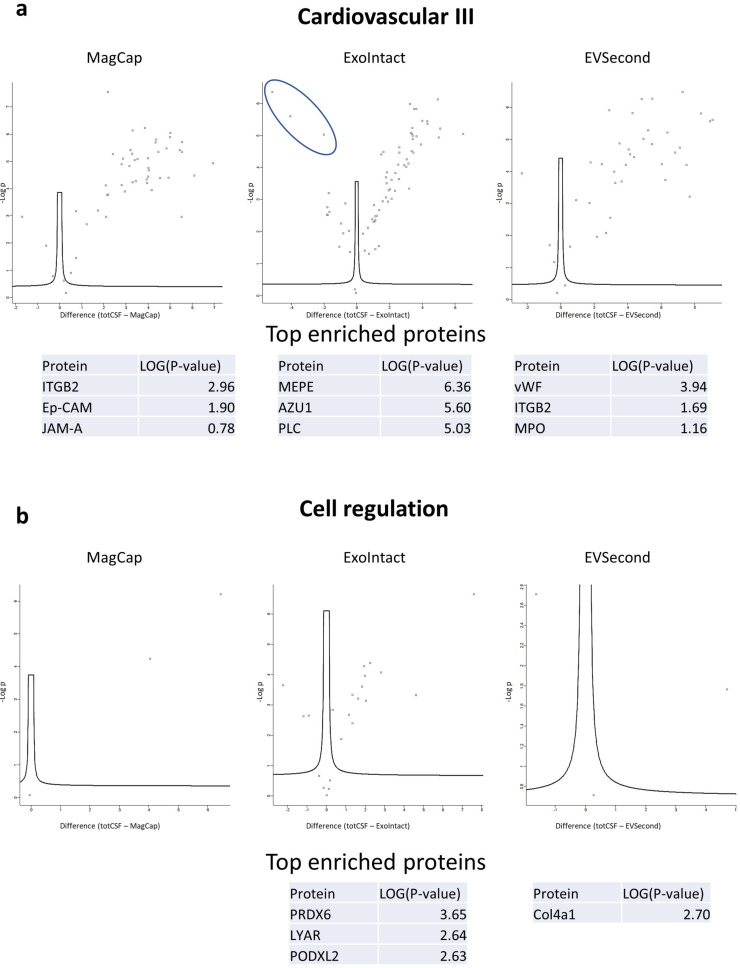
Volcano plots comparing EV samples to total CSF samples. Volcano plots indicate the fold-change differences on the horizontal axis and the *p*-value (double-sided *t*-test with a false discovery rate of 0.05). The plots represent data from the cardiovascular III panel (**a**) and from the cell regulation panel (**b**). The top three enriched proteins are listed. The blue circle in the top middle panel indicates the top three enriched proteins. Tot CSF: total CSF, MagCap: MagCapture.

Finally, we prepared volcano plots to visualize differences between EV and total CSF samples. For the cardiovascular III panel, the MagCap samples showed three proteins that were enriched. The ExoIntact samples had 14 proteins that were enriched, while the EVSecond samples had three (Figure 7a). Regarding the cell regulation panel, neither MagCap nor EVSecond samples demonstrated any upregulated proteins compared to total CSF. For ExoIntact, however, three proteins were upregulated: PRDX6, LYAR, and PODXL2 (Figure 7b). The complete list of proteins and statistical data are provided as Supplementary Materials.

## 3. Discussion

Given the general lack of biomarkers for CNS diseases, we here aimed to develop a novel methodological platform consisting of PEA of CSF-derived EVs. Since this is, to our knowledge, the first time that PEA has been applied to these kinds of samples, we were not sure which isolation method would be suitable. We here compared four different commercially available isolation kits. There were considerable differences between the groups in terms of particle yield and size, with exoEasy isolating both more and larger particles. This is consistent with previous reports that indicate differences in size and yield depending on isolation kit [29,30]. All EV samples showed positivity for the commonly detected EV markers CD63 and CD81. Albumin, a protein that commonly is co-isolated with EVs and represents a contaminant, was greatly reduced in all samples, but the exoEasy samples had the largest concentration.

Given its high sensitivity, we decided to analyze our samples by PEA. Undiluted samples were tested in triplicate; 1:10 and 1:100 dilutions were also included (one replicate per sample group) in order to identify any high-dose hook effects. The neat exoEasy samples did not meet the quality control standard; however, diluted samples did, which likely is due to the hampering effects of the proprietary elution buffer. A buffer exchange step might be necessary to analyze exoEasy samples by PEA.

Although we could not find any previous reports regarding the analysis of CSF-derived EVs by PEA, plasma-derived “neuronal exosomes” were recently analyzed using neurology and neuro-exploratory panels. The authors could distinguish patients with HIV-associated neurocognitive disorder from HIV patients without cognitive impairment [31].

In this study, we could detect a rather large variation in the number of detected proteins between different kits. Unsupervised hierarchical clustering is a common method to reveal subpopulation structures in a dataset and has gained increasing attention for analysis of comprehensive biological datasets in the last two decades [32]. The method builds hierarchies of clusters and has been used to identify clinically relevant biomarkers for several diseases, for example, breast cancer [33]. Interestingly, using unsupervised hierarchical clustering, the samples were correctly grouped by their respective isolation kits. The intensity profiling depicted as heatmaps clearly indicated the differences in the signal based on which isolation kit was used.

The highest number of detected proteins was in the ExoIntact group. In addition, the signal of detected proteins varied depending on the isolation kit, as depicted in Supplementary Figure S1. This highlights the importance of accurate reporting of the EV isolation methodology and the difficulties that might arise when comparing one EV sample set with another, especially if they were isolated using different kits.

For most proteins, the signal was stronger in total CSF compared with EV samples. However, several proteins were upregulated in EVs. During the sample preparation, the EVs were concentrated 100 times using 100 kDa spin filters. If the proteins were large enough (over 100 kDa), they could also be enriched by the concentration step. However, several of the enriched proteins were smaller than 100 kDa; for example, MEPE (~60 kDa), Azu1 (~16–27 kDa), and ITGb2 (~85 kDa). This strengthens the hypothesis that the proteins were concentrated as EV cargo rather than as free, soluble proteins. Several detected proteins in the CSF EVs were “CNS-enriched”, for example PODXL2, OMG, and SLITRK2, which supports the idea that these EVs reflect CNS.

One of the most important questions is whether analyzing CSF EVs instead of total CSF is of value. In this study, we can confidently say that there are differences between the CSF EVs and total CSF, but if this adds diagnostic value or not is still unknown and will be addressed in an upcoming study.

In conclusion, we have here for the first time analyzed CSF-derived EVs using PEA. We clearly demonstrated how different isolation kits affect results, both in terms of the number of identified proteins and signal strengths. In this setting, isolation using ExoIntact resulted in the highest number of identified proteins in both the cardiovascular III and cell regulation panels.

## 4. Materials and Methods

### 4.1. Extracellular Vesicle Isolation

Pooled, commercially available CSF from healthy donors was acquired from PrecisionMed Inc. (Solana Beach, CA, USA) and Lee BioSolutions Inc. (Maryland Heights, MO, USA). The samples were used after approval from the institutional review board of the Shonan Research Center at the Takeda Pharmaceutical Company (CS-00001128 (approved 10 February 2016) and CS-00200049 (approved 11 July 2019)). The CSF was cleared by centrifugation: 2000× *g*, 4 °C for 5 min in the first step, after which the supernatants were then transferred to new tubes and subjected to a second centrifugation step of 10,000× *g*, 4 °C for 20 min. The supernatant was collected as cleared CSF, aliquoted (per kit: 2 × 1100 µL and 1 × 1210 µL), and stored at −80 °C. EVs were isolated using exoEasy (Qiagen, Hilden, Germany), EVSecond L70 (GL Sciences, Tokyo, Japan), ExoIntact (HMT Biomedical, Yamagata, Japan), and MagCapture (Fujifilm Wako Pure Chemical Corporation, Osaka, Japan) according to the manufacturers’ instructions. Prior to isolation with MagCapture, the cleared CSF was concentrated using ultrafiltration spin filters (Vivacon 500, Sartorius, Göttingen, Germany) to reach a volume of less than 1000 µL. After isolation, the EV suspensions from each kit were concentrated using Vivacon 500 spin filters to volumes less than 11 µL, and PBS was subsequently added to reach a final volume of 1/100 of the starting cleared CSF volume (per kit: 2 × 11 µL and 1 × 12.1 µL). One microliter from each sample was saved, and 1.1 µL of the 12.1 µL samples was used to produce 1:10 and 1:100 diluted samples (10 µL each). In total, 26 samples were analyzed using PEA (Olink Proteomics Inc, Uppsala, Sweden): 3 × nondiluted samples from each kit (representing three independent isolations), 1 × 1:10 and 1 × 1:100 diluted samples from each kit, 3× total cleared CSF, and 1 × 1:10 and 1:100 diluted samples from total cleared CSF. The 10 µL samples were mixed with 13 µL M-PER (Thermo Fischer Scientific, Waltham, MA, USA) with a 1:100 protease inhibitor cocktail (Cell Signaling Technology, Danvers, MA, USA). A lysis buffer with protease inhibitor mixed with PBS at the same final concentration as the samples was included as a negative control. The samples were sequentially added in a random order to a 96-well plate (AB Gene PCR full skirt, Thermo Fischer Scientific, Waltham, MA, USA), sealed with a temperature-resistant plate-sealer, and stored at −80 °C. The samples were shipped on dry ice and analyzed at the Olink Proteomics Inc Boston site, MA, USA.

### 4.2. Nanoparticle Tracking Analysis

EVs were analyzed using nanoparticle tracking analysis (NanoSight NS500Z, Malvern Panalytical, Malvern, UK). For each sample, five videos were recorded and analyzed using Nanoparticle Tracking Analysis software version 2.3 build 0033 (Malvern Panalytical, Malvern, UK).

### 4.3. ELISA

CD63 and CD81 were measured using PS Capture (Fujifilm Wako Pure Chemical Corporation, Osaka, Japan) according to the manufacturer’s instructions. The CD63 antibody was provided with the kit and used at 1:100 dilution. The CD81 antibody (M38, Novus Biologicals, Littleton, CO, USA) was used at 1:200 dilution. The exoEasy samples were diluted 1:5, while the other EV samples were diluted 1:2.5.

Albumin concentration was measured using an ELISA kit (E88-129 Bethyl Laboratories, Montgomery, TX, USA) according to the manufacturer’s instructions. The exoEasy samples were diluted 1:200, the other EV samples at 1:50, and total CSF at 1:2000.

### 4.4. Data Analysis

Initial data clean-up was performed in Microsoft Excel 360. Samples and values that did not meet Olink’s quality control criteria were excluded. We next evaluated high-dose hook effect artifacts and removed markers that had an 80% or higher signal in 1:10 or 1:100 dilutions compared with nondiluted samples.

The freely available software platform Perseus v1.6.7.0 [34] was used for subsequent analysis of the cleaned PEA data. Profile plots, histograms, multiscatter plots, hierarchical clustering, principal component analysis, and volcano plots were made using the software’s default settings.

Venn diagrams (Figure 3e,f) were produced using the functional enrichment analysis tool [35].

Bar charts and statistical analyses were made using Graph Pad Prism version 8 (GraphPad Software, CA, USA). Error bars indicate standard deviations. Student’s *t*-test was used and statistical significance indicated as: * *p* ≤ 0.05, ** *p* ≤ 0.01, and *** *p* ≤ 0.001.

## Supplementary Materials

Supplementary Materials can be found at https://www.mdpi.com/1422-0067/21/24/9425/s1.

## Abbreviations

AD	Alzheimer’s disease
BBB	Blood–brain barrier
CNS	Central nervous system
CSF	Cerebrospinal fluid
EV	Extracellular fluid
PCA	Principal component analysis
PEA	Proximity extension assay
