# Supplementary material for: Analysis of Cerebrospinal Fluid Extracellular Vesicles by Proximity Extension Assay: A Comparative Study of Four Isolation Kits
Source: Int J Mol Sci. 2020 Dec 10;21(24):9425. doi: 10.3390/ijms21249425 (PMC7763352; doi:10.3390/ijms21249425)
Supplement: Supplementary file 1 [file ijms-21-09425-s001.pdf]

International Journal of
Molecular Sciences

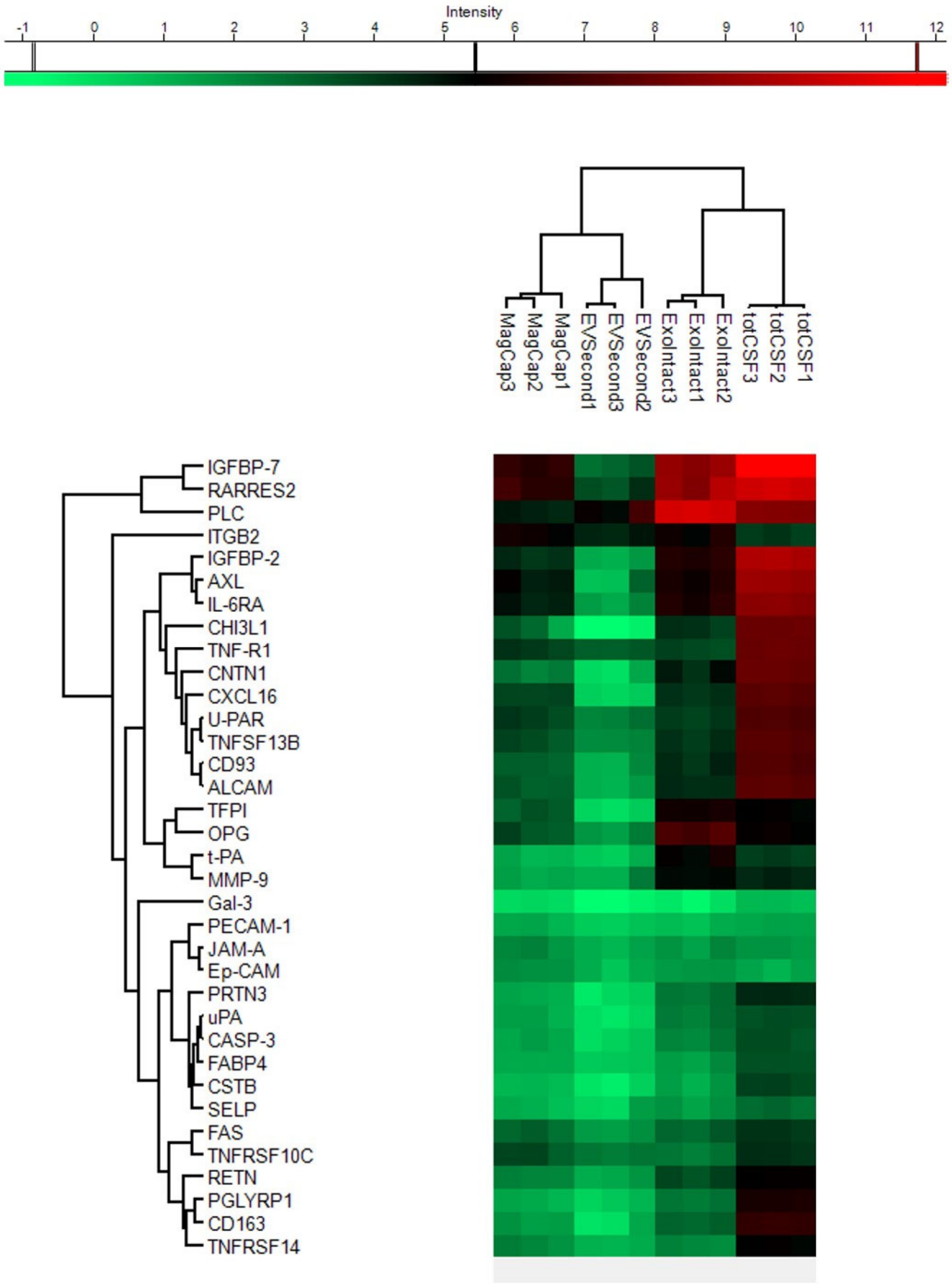

Supplementary Figure S1. Hierarchical clustering of data from the cardiovascular III panel, including only markers detected in all groups. Each column represents a sample, and each row represents an analyte. The color of each cell represents intensity, as indicated by the color gradient on the top of the image; gray color indicates a missing value. Tot CSF: total CSF, MagCap: MagCapture.
